# Conditions of gestation, childbirth and childhood associated with C-peptide in young adults in the 1982 Birth Cohort in Pelotas-RS; Brazil

**DOI:** 10.1186/s12872-017-0613-3

**Published:** 2017-07-11

**Authors:** Romildo Luiz Monteiro Andrade, Denise Petrucci Gigante, Isabel Oliveira de Oliveira, Bernardo Lessa Horta

**Affiliations:** 1 Hospital Universitário Cassiano Antonio de Moraes, Av. Mal. Campos, 1355 - Santa Cecilia, Vitória, ES 29043-260 Brazil; 2 Programa de Pós-Graduação em Epidemiologia da Universidade Federal de Pelotas (UFPel), Centro de Epidemiologia Dr. Amilcar Gigante, R. Mal. Deodoro, 1160 - Centro, Pelotas, Vitória, RS 96020-220 Brazil; 3 Dante Michelinne 2431, apto 303, Mata da Praia, Vitória, ES CEP: 29066-430 Brazil

**Keywords:** Pregnancy, Parturition, Growth, C-peptide, Young adults

## Abstract

**Background:**

The connecting peptide in insulin has been associated with cardiovascular risk and overall mortality in the adult population. However, its early determinants are unknown. Assess the association of exposures during pregnancy, delivery, and childhood with C-peptide among 22–23 years old individuals prospectively followed since birth, in a southern Brazilian city.

**Methods:**

In 1982, all hospital births in the city were identified and those livebirths whose families lived in the urban area were evaluated (*n* = 5914). The 1982 Pelotas Birth Cohort has prospectively followed these subjects at different moments. In this study, we evaluated the association of C-peptide with exposures occurring during pregnancy, delivery and childhood. In the 22–23 years follow-up visit, we tried to follow the whole cohort and the subjects were interviewed, examined and donated a blood sample. C-peptide was measured using the chemiluminescence immunoassay technique (Immulite®–Siemens, Germany).

**Results:**

In the 22–23 years visit, 4297 subjects were interviewed and the C-peptide was measured in 3807. The geometric mean of C-peptide was 0.83 ng/mL and the mean was higher among women. In the adjusted analysis, C-peptide was positively associated with family income at birth, lower among children of non-white mothers (0.90; CI95% 0.84–0.96), higher among females (1.22; CI95% 1.16–1.28), and positively associated with rapid weight gain between two and four years of age (1.18; CI95% 1.05–1.32).

**Conclusion:**

Family income at birth, non-white maternal skin color, and rapid weight gain between two and four years of age were associated with high levels of C-peptide.

**Electronic supplementary material:**

The online version of this article (doi:10.1186/s12872-017-0613-3) contains supplementary material, which is available to authorized users.

## Backrgound

In 2012, cardiovascular diseases were the main cause of death worldwide [1], accounting for 17.5 million deaths or 46.2% of the deaths due to non-communicable diseases [2, 3]. It is estimated that, by 2020, the higher prevalence of risk factors, such as obesity, diabetes, and dyslipidemia [4] will lead to an increase in ischemic heart disease by up to 30% among women and 60% among men in high-income countries and by 120% and 137% in mid- and low-income countries, respectively [5].

Secreted by β-cells from isles of Langerhans, the connecting peptide, called C-peptide, is released at equimolar amounts as insulin [6]. It was initially considered an inert substance in the pro-insulin molecular chain. But, evidence suggests that C-peptide may play a role at the cell-membrane level, including the endothelium and kidney cells [7–10].

Vasic et al. pointed that C-peptide would have pro-inflammatory effects on different tissues, such as blood vessels and kidney glomeruli [11]. Moreover, Ronnemaa et al. found that coronary artery disease in insulin-dependent diabetics was associated with high C-peptide levels (≥ 0.20 nmol/L) compared with non-diabetics [12]. Donatelli et al. reported higher C-peptide levels among obese hypertensive diabetics compared to obese and obese-hypertensive individuals [13]. Cabrera de Leon et al. reported that among persons with insulin resistance, C-peptide would be positively associated with the risk of myocardial infarction (RR 2.8; CI95% 1.1–6.9) and coronary artery disease (RR 2.4; CI95% 1.3–4.6) [14]. These studies have not been able to evaluate whether C-Peptide is a marker of insulin resistance or an independent cardiovascular risk factor.

Studies on life cycle epidemiology have reported that exposures during gestation or in the first years of life would be associated with human capital and the development of non-communicable chronic diseases in adulthood [15–20]. To our knowledge, the association of early exposures with C-peptide has not been evaluated.

The present study was aimed at assessing the association of exposures during pregnancy, delivery, and childhood with C-peptide among 22–23 years old individuals who have been prospectively followed since birth, in a southern Brazilian city.

## Methods

In 1982, the maternity hospitals in Pelotas, RS, Brazil, were visited daily and all births were identified. The live births whose families lived in the urban area of the city (*n* = 5914) were examined and their mothers interviewed. The 1982 Pelotas Birth Cohort has prospectively followed these individuals at different ages [21, 22]. Between October 2004 and August 2005, an attempt was made to follow all cohort members, who were interviewed at home and invited to visit the laboratory for collection of blood samples. In the present study, we included all subjects who donated a blood sample.

The association of the following exposures related to gestation, delivery, and childhood with the outcome (C-peptide at 22–23 years) was evaluated:

- family income at birth in multiples of minimum wage;
- maternal education at birth in completed years of schooling;
- sex;
- maternal age at birth;
- maternal skin color;
- maternal weight gain during pregnancy (estimated from pregestational weight and weight at childbirth), categorized as adequate or inadequate according to the parameters of the Institute of Medicine [23];
- maternal smoking during pregnancy;
- maternal morbidity during pregnancy (gestational diabetes and hypertension);
- type of delivery (vaginal and cesarean section);
- birthweight, measured by the hospital staff with pediatric scales calibrated
- weekly by the research team;
- breastfeeding duration, assessed in the visits at two and four years of age. The
- present study used the information closest to the age at weaning;
- weight gain rate between 0 and 2 and 2–4 years old, assessed based on changes in weight for age Z-score. An increase equal to or above 0.67 standard deviations (SD) was employed to define the occurrence of rapid weight gain [24].

C-peptide was measured using the chemiluminescence immunoassay technique.

(Immulite®–Siemens, Germany) [25, 26].

The statistical analysis was performed in the software Stata version 13.1. Since the C-peptide distribution was asymmetrical, the variable was transformed into logarithm and the geometric mean was obtained from the inverse transformation of its logarithm. The multiple linear regression followed a conceptual model with five hierarchical levels. The first level features the sociodemographic variables: family income, maternal education, maternal age, maternal skin color, and sex. The second level included maternal smoking and weight gain during pregnancy, the third level included the occurrence of diabetes and hypertension during pregnancy, and the fourth level included the variables related to delivery and birth conditions, type of delivery, birthweight, and intrauterine growth restriction. The fifth level included weight gain between birth and two years and between two and four years of age. At each hierarchical level, backward selection was carried out and the variables with *p* < 0.20 were maintained in the model. As the blood samples were collected at random and because the time of fasting is associated with C-peptide, all analyses were adjusted to time of fasting of each participant. Later, residual analyses were performed in order to check for normality, homoscedasticity, and independence of terms.

The study was approved by the Research Ethics Committee of the Medical School of the Federal University of Pelotas (UFPel) under protocol no. OF. 16/12 and the interviews and blood collections were carried out after the participants provided written consent.

## Results

In the 2004–5 visit, 4297 individuals were interviewed, which added to the 282 deaths identified among the participants of the cohort, represented a follow-up rate of 77.4% of the original cohort. C-peptide was assessed in 3807 subjects. Table 1 describes the population studied according to its socioeconomic characteristics and conditions related to gestation and childhood, 50.4% of the families earned between 1.1 and 3.0 minimum wage, 43.3% of the mothers had between five and eight years of schooling, 82% reported being white, 35% smoked during pregnancy, 0.3% had diabetes and 5.4% had hypertension during pregnancy. With respect to birthweight, 7% were low birthweight. About one third of the participants had rapid weight gain in the first two years of life and 5%, between two and four years of age.

**Table 1:** Participants’ characteristics

Variables studied	Study participants	
	*n*	%
Sociodemographic conditions		
Income (multiples of minimum wage)		
<1	769	20.3
1.1–3.0	1908	50.4
3.1–6.0	714	18.8
> 6.0	39	10.5
Mother’s education (years)		
0–4	1266	33.3
5–8	1645	43.3
9–11	410	10.7
≥ 12	481	12.7
Gestation conditions		
Mother’s age (years)		
< 20	549	27.9
20–29	2213	48.9
≥ 30	1045	23.2
Mother’s skin color		
White	3114	81.8
Non-white	692	18.2
Maternal smoking		
Yes	1347	35.4
No	2460	64.6
Maternal diabetes		
Yes	13	0.3
No	3794	99.7
Maternal hypertension		
Yes	216	5.4
No	3588	94.6
Maternal weight gain		
Insufficient	960	29.8
Adequate	1188	36.9
Excessive	1070	33.3
Childbirth conditions		
Type of childbirth		
Vaginal	2756	72.4
Cesarean section	1051	27.6
Birthweight (grams)		
< 2500	267	7.0
2500–2999	917	24.1
3000–3499	1449	38.1
≥ 3500	1173	30.8
Intrauterine growth restriction		
AGA	2590	85.2
SGA	448	14.8
Childhood conditions		
Breastfeeding (months)		
< 1	801	21.8
1–2.9	958	26.0
3–5.9	834	22.6
≥ 6	1091	29.6
Increased weight gain rate (0–2 years old)		
Yes	973	34.5
No	1845	65.5
Increased weight gain rate (2–4 years old)		
Yes	157	4.8
No	3104	95.2

*SGA* Small for the gestational age, *AGA* Adequate for the gestational age

Table 2 shows the geometric mean of C-peptide according to the exposure variables. Because sex did not modify the associations, the analyses were not stratified by sex. The geometric mean of C-peptide was positively associated with family income (*p* < 0.001). Regarding maternal education, the mean C-peptide values increased until the group with nine to 11 years of schooling (0.89; CI95% 0.83–0.96) and was lower among the children of non-white mothers (0.73; CI95% 0.69–0.77).

**Table 2:** Geometric mean of C-peptide according to the exposure variables

Variables	C-peptide geometric mean	CI95%	*p* value
Sex			< 0.001
Male	0.76	0.73–0.78	
Female	1.19	1.13–1.25	
Income (multiples of minimum wage)			< 0.001*
< 1	0.76	0.72–0.80	
1.1–3.0	0.81	0.86–0.84	
3.1–6.0	0.90	0.85–0.95	
> 6.0	0.88	0.81–0.95	
Mother’s education (years)			0.024
0–4	0.79	0.76–0.82	
5–8	0.83	0.79–0.85	
9–11	0.89	0.83–0.96	
≥ 12	0.85	0.79–0.91	
Mother’s age (years)			0.872
< 20	0.81	0.77–0.87	
20–29	0.82	0.80–0.85	
≥ 30	0.83	0.80–0.87	
Mother’s skin color			< 0.001
White	0.85	0.83–0.87	
Non-white	0.73	0.69–0.77	
Maternal smoking			0.925
Yes	0.83	0.79–0.86	
No	0.83	0.80–0.85	
Maternal diabetes			0.503
Yes	0.72	0.40–0.77	
No	0.83	0.81–0.85	
Maternal hypertension			0.909
Yes	0.83	0.75–0.91	
No	0.82	0.80–0.84	
Maternal weight gain during pregnancy			0.349
Insufficient	0.83	0.77–0.84	
Adequate	0.82	0.74–0.87	
Excessive	0.86	0.80–0.88	
Type of childbirth			0.616
Vaginal	0.82	0.80–0.84	
Cesarean section	0.83	0.79–0.87	
Birthweight (grams)			0.897
< 2500	0.81	0.74–0.89	
2500–2999	0.82	0.78–0.86	
3000–3499	0.83	0.80–0.86	
≥ 3500	0.82	0.78–0.85	
Intrauterine growth restriction			0.135
Yes	0.80	0.74–0.85	
No	0.84	0.81–0.86	
Breastfeeding (months)			0.557
< 1	0.81	0.76–0.85	
1–2.9	0.84	0.80–0.88	
3–5.9	0.81	0.77–0.85	
≥ 6	0.83	0.79–0.87	
Rapid growth (0–2 years old)			0.238
Yes	0.82	0.79–0.85	
No	0.85	0.81–0.89	
Increased weight gain rate (2–4 years old)			0.124
Yes	0.83	0.81–0.85	
No	0.91	0.80–1.03	

*Kruskal-Wallis

Table 3 shows the adjusted analyses. In the first hierarchical level, family income at birth, skin color and sex remained in the model. Family income was positively associated with C-peptide. Children of non-white mothers had lower C-peptide levels (0.90; CI95% 0.84–0.96), while females had higher C-peptide mean (1.22; CI95% 1.16–1.28).

**Table 3:** Analysis of the independent variables and C-peptide adjusted for fasting time

Hierarchical level	Variables	Analysis 1				Analysis 2			
		*n*	β	CI95%		*n*	β	CI95%	
HL I	Income (multiples of minimum wage)	3660				3139			
	< 1		1.00				1.00		
	1.1–3.0		1.04	0.98	1.10		1.03	0.96	1.09
	3.1–6.0		1.14	1.06	1.23		1.12	1.03	1.22
	> 6.0		1.12	1.03	1.23		1.07	0.98	1.19
	Mother’s education (years)	3673							
	< 4		1.00						
	5–8		1.03	0.97	1.09				
	9–11		1.09	1.00	1.18				
	≥ 12		1.05	0.97	1.13				
	Mother’s age (years)	3678							
	< 20		1.00						
	20–29		1.00	0.93	1.07				
	≥ 30		1.01	0.94	1.09				
	Mother’s skin color	3677							
	White		1.00				1.00		
	Non-white		0.88	0.83	0.94		0.90	0.84	0.96
	Neonate sex	3678							
	Male		1.00				1.00		
	Female		1.20	1.14	1.25		1.22	1.16	1.28
HL II	Maternal smoking	3678							
	No		1.00						
	Yes		1.01	0.96	1.06				
	Maternal weight gain during pregnancy	3109							
	Adequate		1.00						
	Insufficient		0.99	0.93	1.05				
	Excessive		1.02	0.96	1.09				
HL III	Maternal diabetes	3678							
	No		1.00						
	Yes		0.84	0.57	1.24				
	Maternal hypertension	3675							
	No		1.00						
	Yes		1.00	0.91	1.11				
HL IV	Type of childbirth	3678							
	Vaginal		1.00						
	Cesarean section		1.01	0.96	1.06				
	Intrauterine growth restriction	3678							
	No		1.00						
	Yes		0.98	0.91	1.06				
	Birthweight (grams)	3677							
	< 2500		1.00						
	2500–2999		0.99	0.90	1.10				
	3000–3499		0.99	0.91	1.10				
	≥ 3500		0.97	0.88	107				
HL V	Breastfeeding (months)	3560							
	< 1		1.00						
	1–2.9		1.02	0.95	109				
	3–5.9		0.99	0.92	1.08				
	≥ 6		1.03	0.97	1.10				
	Increased weight gain rate (0–2 years old)	2718							
	No		1.00						
	Yes		1.04	0.98	1.10				
	Increased weight gain rate (2–4 years old)	3152							
	No		1.00				1.00		
	Yes		1.13	1.01	1.27		1.18	1.05	1.32

Multiple linear regression. Analysis 1: independent variable; C-peptide and fasting time. Analysis 2: Hierarchical level I (HL I): adjusted for sociodemographic variables and fasting time; HL II to V: hierarchically adjusted for variables selected from the previous level and other variables of subsequent levels and fasting time

**Fig. 1:**
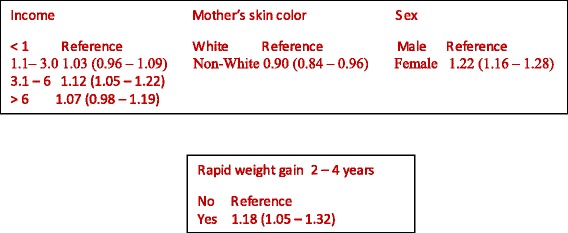
Hierarchical model for C-peptide

The variables in the second (maternal smoking and maternal weight gain during pregnancy), third (maternal diabetes and hypertension during pregnancy), and fourth (type of delivery, intrauterine growth restriction, birthweight) levels did not reach the significance level (*p* < 0.20) to remain in the model. After adjusting for the variables at a higher hierarchical level, rapid weight gain between two and four years was positively associated with C-peptide (1.18; CI95% 1.05–1.32), while birthweight and weight gain in the first two years of life were not associated with C-peptide Fig. 1.

## Discussion

In a cohort followed since birth in a city in southern Brazil, family income at birth was directly associated with C-peptide at 22-23 years old. Mean C-peptide was higher among women, among those born in families with income between three and six times the minimum wage, and among the children of white mothers. Increased weight gain rate between two and four years old was also positively associated with serum C-peptide levels, while birthweight and weight gain in the first two years of life had no association.

Given the long follow-up period, the low percentages of losses, and the fact there were no great differences in the follow-up rates according to socioeconomic characteristics or gestation and birth, the possibility of selection bias is small (Additional file 1: Table S1). Moreover, the information on the exposures was collected in childhood, close to the events, which lowers the possibility of error in information collection and the possibility of error in the nondifferential classification.

On the other hand, C-peptide was measured in randomly collected blood samples. In order to keep a possible association between fasting time and the exposures of interest from introducing bias into the association measures, the analyses were adjusted for fasting time.

C-peptide levels were positively associated with family income at birth, suggesting that exposure to a higher socioeconomic level during gestation is related to higher C-peptide levels in adulthood. This association can be explained by differences in exposure to contemporary risk factors, such as diet. Another study on this same cohort observed that the consumption of ultra-processed foods was higher among individuals with higher socioeconomic level, which, in turn, would be associated with higher risk of obesity [27].

The children of non-white mothers had lower C-peptide levels. Such findings may derive from the lower socioeconomic level among nonwhite individuals [28]. Aiming to investigate the possibility of confound by income in the association between maternal skin color and C-peptide, the analyses were adjusted for family income. However, even after adjustment, C-peptide levels were lower among the children of non-white mothers (0.90; CI95% 0.85-0.96), which suggests that skin color independently influences family income in the determination of serum C-peptide levels.

In the present study, women had the highest C-peptide means in adulthood, contrasting with the results by Li et al., who reported similar means between the sexes [29].

Increased weight gain rate between two and four years old was associated with higher serum C-peptide levels in adulthood. A study on the same cohort showed that increased weight gain rate in the first years old life reduces morbidity-mortality among small-for-the-gestational-age (SGA) children [30]. On the other hand, other authors reported that rapid recovery of growth may increase the risk of developing cardiometabolic diseases in adulthood [31–33]. However, a study based on data from five cohorts in developing countries observed that the relative weight gain in the first four years of life was associated with higher risk of overweight and high arterial blood pressure in adulthood, but not with plasma glycemia [34]. The present findings, however, indicate that early growth would not be associated with C-peptide in adulthood, while rapid growth between two and four years old would increase C-peptide at 22-23 years old, which suggests that late increased weight gain rate in childhood would impact the glucose-insulin metabolism in adulthood.

## Conclusion

The results in the present study suggest that adults from families with income above three times the minimum wage, children of non-white mothers, and those who had increased weight gain rate between the second and fourth years of life had higher serum C-peptide levels at 22-23 years old. Further studies may contribute to confirming.

## Additional file

Additional file 1: Table S1. Characteristics of the participants in the 1982 cohort and sample of individuals analyzed in the study. (DOC 67 kb)
