# Supplementary material for: Conditions of gestation, childbirth and childhood associated with C-peptide in young adults in the 1982 Birth Cohort in Pelotas-RS; Brazil
Source: BMC Cardiovasc Disord. 2017 Jul 11;17:181. doi: 10.1186/s12872-017-0613-3 (PMC5504841; doi:10.1186/s12872-017-0613-3)
Supplement: Additional file 1: Table S1. — Characteristics of the participants in the 1982 cohort and sample of individuals analyzed in the study. (DOC 67 kb) [file 12872_2017_613_MOESM1_ESM.doc]

| **Table S1. Characteristics of the participants in the 1982 cohort and sample of individuals analyzed in the study.** | | |  |
| --- | --- | --- | --- |
|  | Participants in 1982 | Study participants |  |
|  | n (%) | n (%) | |
| **Sociodemographic conditions** |  |  |  |
| Income (multiples of minimum wage) * | 5,885 | 3788 |  |
| < 1 | 1,288 (21.9) | 769 (20.3) |  |
| 1.1-3.0 | 2,789 (47.4) | 1,908 (50.4) |  |
| 3.1-6.0 | 1,091 (18.5) | 714 (18.80) |  |
| > 6.0 | 717 (12.2) | 397 (10.5) |  |
| Mother’s education (years) | 5,907 | 3,802 |  |
| 0-4 | 1,960 (33.2) | 1,266 (33.3) |  |
| 5-8 | 2,454 (41.5) | 1,645 (43.0) |  |
| 9-11 | 654 (11.1) | 410 (10.78) |  |
| ≥ 12 | 839 (14.2) | 481 (12.6) |  |
| **Gestation conditions** |  |  |  |
| Mother’s age (years) | 4,254 | 3,807 |  |
| < 20 | 912 (15.4) | 549 (27.9) |  |
| 20-29 | 3,342 (58.2) | 2,213 (48.9) |  |
| ≥ 30 | 1,559 (26.4) | 1,045 (23.1) |  |
| Mother’s skin color | 5,911 | 3,804 |  |
| White | 4,851 (82.1) | 3,114 (81.8) |  |
| Non-white | 1,060 (17.9) | 692 (18.2) |  |
| Maternal smoking | 5,914 | 3,807 |  |
| Yes | 2,103 (35.6) | 1,347 (35.4) |  |
| No | 3,811 (64.4) | 2,460 (64.6) |  |
| Maternal diabetes | 5914 | 3,807 |  |
| Yes | 18 (0.3) | 13 (0.3) |  |
| No | 5,896 (99.7) | 3,794 (99.7) |  |
| Maternal hypertension | 5,910 | 4,010 |  |
| Yes | 315 (5.3) | 216 (5.39) |  |
| No | 5,595 (94.7) | 3,794 (94.61) |  |
| Maternal weight gain^†^ | 4,976 | 3,218 |  |
| Insufficient | 1,787 (35.9) | 960 (29.8) |  |
| Adequate | 1,515 (30.5) | 1,188 (36.9) |  |
| Excessive | 1,674 (33.6) | 1,070 (33.3) |  |
| **Childbirth conditions** |  |  |  |
| Type of childbirth | 5,914 | 3,807 |  |
| Vaginal | 4,282 (72.4) | 2,756 (72.39) |  |
| Cesarean section | 1,632 (27.6) | 1,051 (27.61) |  |
| Birthweight (grams) | 5,909 | 3,806 |  |
| < 2.500 | 534 (9.0) | 267 (7.0) |  |
| 2,500-2,999 | 1,393 (23.6) | 917 (24.1) |  |
| 3,000-3,499 | 2,220 (37.6) | 1,449 (38.1) |  |
| ≥ 3,500 | 1,762 (29.8) | 1,173 (30.8) |  |
| Intrauterine growth restriction^‡^ | 4,670 | 3,038 |  |
| AGA | 3,977 (67.2) | 2,590 (68.0) |  |
| SGA | 693 (11.7) | 448 (11.8) |  |
| **Childhood conditions** |  |  |  |
| Breastfeeding (months) | 5,332 | 3,684 |  |
| < 1 | 1,171 (22.0) | 801 (21.7) |  |
| 1-2.9 | 1,405 (26.3) | 958 (26.0) |  |
| 3-5.9 | 1,212 (22.7) | 834 (22.6) |  |
| ≥ 6 | 1,544 (29.0) | 1,091(29.6) |  |
| Increased weight gain rate (0-2 years old)^§^ | 3,916 | 2,818 |  |
| Yes | 1,336 (34.0) | 973 (34.5) |  |
| No | 2,580 (66.0) | 1,845 (65.5) |  |
| Increased weight gain rate (2-4 years old)// | 4,513 | 3,261 |  |
| Yes | 209 (5.0) | 157 (4.8) |  |
| No | 4,304 (95.0) | 3,104 (95.2) |  |
| Missing information - *19; †589; †1244; §989; //546  SGA: Small for the gestational age; AGA: Adequate for the gestational age | | |  |
